# The Aerodynamics and Energy Cost Assessment of an Able-Bodied Cyclist and Amputated Models by Computer Fluid Dynamics

**DOI:** 10.3390/medicina56050241

**Published:** 2020-05-18

**Authors:** Pedro Forte, Daniel A. Marinho, Ricardo Silveira, Tiago M. Barbosa, Jorge E. Morais

**Affiliations:** 1 Department of Sports, Higher Institute of Educational Sciences of the Douro, 4560-708 Penafiel, Portugal; ricardo.silveira@iscedouro.pt (R.S.); morais.jorgestrela@gmail.com (J.E.M.); 2 Departamento de Desporto e Educação Física, Instituto Politécnico de Bragança, 5300-253 Bragança, Portugal; barbosa@ipb.pt; 3 Research Center for Sports Health and Human Development, 6201-001 Covilhã, Portugal; 4 Department of Sports Sciences, University of Beira Interior, 6201-001 Covilhã, Portugal; marinho.d@gmail.com

**Keywords:** CFD, energy cost, amputee, cyclists, comparison

## Abstract

*Background and Objectives:* The aim of this study was to assess and compare the drag and energy cost of three cyclists assessed by computational fluid dynamics (CFD) and analytical procedures. *Materials and methods:* A transradial (Tr) and transtibial (Tt) were compared to a full-body cyclist at different speeds. An elite male cyclist with 65 kg of mass and 1.72 m of height volunteered for this research with his competition cloths, helmet and bicycle with 5 kg of mass. A 3D model of the bicycle and cyclist in the upright position was obtained for numerical simulations. Upon that, two more models were created, simulating elbow and knee-disarticulated athletes. Numerical simulations by computational fluid dynamics and analytical procedures were computed to assess drag and energy cost, respectively. *Results:* One-Way ANOVA presented no significant differences between cyclists for drag (*F* = 0.041; *p* = 0.960; *η*^2^ = 0.002) and energy cost (*F* = 0.42; *p* = 0.908; *η*^2^ = 0.002). Linear regression presented a very high adjustment for absolute drag values between able-bodied and Tr (*R*^2^ = 1.000; *Ra*^2^ = 1.000; *SEE* = 0.200) and Tt (*R*^2^ = 1.00; *Ra*^2^ = 1.000; *SEE* = 0.160). The linear regression for energy cost presented a very high adjustment for absolute values between able-bodied and Tr (*R*^2^ = 1.000; *Ra*^2^ = 1.000; *SEE* = 0.570) and Tt (*R*^2^ = 1.00; *Ra*^2^ = 1.00; *SEE* = 0.778). *Conclusions:* This study suggests that drag and energy cost was lower in the able-bodied, followed by the Tr and Tt cyclists.

## 1. Introduction

Research on athletes with disability has been requested by different world organizations [1]. In biomechanics, little is known about the differences between athletes with disability and their able-bodied counterparts [1,2]. Most of the methodologies, training assessment protocols and Paralympics testing are based on evidence-based information (research) with able-bodied subjects [3]. Moreover, the majority of strategies to improve Paralympics performance are based on able-bodied protocols [4]. However, based on able-bodied subjects, a bias may occur when extrapolating their results to athletes with disability. Moreover, effort intensities and exertion levels might be different between able-bodied and disabled athletes [5].

Cycling is one of the most popular sports in athletes with disabilities. In this Paralympic cycling, the classifications are split-up into five classes, according to athlete’s condition (WCi, i.e., *i* = 1, 2, 3, 4 or 5 with limitations and or amputations in lower and upper limbs). Amputee participants with bilateral knee amputation and with severe athetose or ataxia compete in C1 and with unilateral knee and moderate athetose or ataxia compete in C2. Uni-lateral knee amputation and bilateral elbow amputation with hemiplegic or diplegic spasticity and/or mild athetosis or ataxia compete in C4 class, whereas, C5 cyclists have unilateral arm amputation and mild monoplegic spasticity [6,7,8]. Moreover, it is possible to compete with and without prosthesis [9]. Cyclists positions have the goal of minimizing the air resistance (i.e., drag) and Paralympic cyclists with amputations mainly adopt the normal or upright position in cycling [6,7,8]. The cyclist’s aerodynamics depends on the body posture on the bicycle and the physical impairment, such as amputations [10,11]. To date, at least two studies assessed and heightened the prosthesis influence on aerodynamics [10,11]. The energy cost of transportation in cycling is drag dependent. Hence, it may differ in accordance with one’s type of amputation [12]. However, no study was founded comparing the able-bodied with transradial (Tr) and transtibial (Tt) amputee cycists.

Cyclists must deliver a certain amount of energy to reach a target speed, and overcome the resistive forces. The energy expenditure may also determine the training or racing effort [13]. Thus, racing will be as intense as the energy expenditure. It is possible to assess the energy expenditure and energy cost by a set of analytical procedures that encompass the measurement of drag and rolling resistance [14,15]. Rolling resistance is dependent on the mass of the bicycle-cyclists system, gravitational acceleration, rolling resistance coefficient and velocity, whereas, drag is dependent on the speed, fluid density, drag coefficient and object/system surface area [16].

Numerical simulations by computational fluid dynamics (CFD) is a valid and reliable method to assess drag, and can provide as outputs the drag coefficient, drag force and the fluid flow around the body [3,17,18,19]. Based on that, it is possible to determine the energy cost of transportation (Ec) based on these CFD outputs. Recently, some researchers have been using different mannequins to assess drag force [20]. Thus, single mannequin to perform the numerical simulations allow to compare edited mannequins/models [20,21,22,23]. Upon that, data about energy cost may help coaches, cyclists and sports analysts to control cyclist’s performance [15]. It is possible to assess drag and rolling resistance by different techniques, such as analytical procedures, coasting decelerations, wind tunnel testing and numerical simulations by CFD [3]. Wind tunnel testing is deemed as the gold-standard to assess drag. Nevertheless, it is considered an expensive procedure that is not readily available to most [3,24]. The CFD allows assessing drag in specific and controlled conditions and shows very good adherence to wind tunnel testing [3,16] and CFD usually assesses a representative participant of a specific group [3,16,19]. Moreover, this methodology avoids confounding factors such as inter-subject variability [3,16,19]. Coasting deceleration techniques do not control environmental factors such as temperature and wind [3,25] and the analytical procedures require a set of assumptions [3,26,27].

To date, no study was found in the literature comparing amputated cyclist’s energy cost with their counterparts based on CFD and analytical procedures. Thus, the aim of this study was to assess and compare three cyclist’s energy cost assessed by CFD and analytical procedures, with Tr and Tt amputation models with an able-bodied at different speeds. It was hypothesized that amputated cyclist present different energy costs in comparison to their able-bodied counterparts.

## 2. Materials and Methods

### 2.1. Participant and Scanning

An elite male cyclist with 65 kg of mass and 1.72 m of height volunteered for this research. The participant wear his competition cloths (with polyester, polyamide, polypropylene and elastane fibers), helmet (LAS, Cronometro) and bicycle with 7 kg of mass (KTM, Revelator Master 2017). The participant was competing in the national level competitions. All the procedures were in accordance with the Helsinki declaration and informed written consent was taken beforehand. The Ethics Committee of the University of Beira Interior under the registration number D1608 granted approval in 2018.

**Figure 1:**
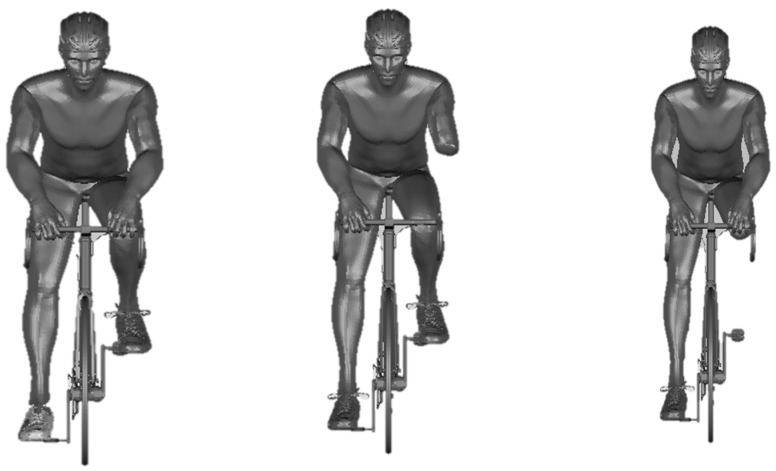
Able-Bodied, Transradial (Tr) and Transtibial (Tt) amputee bicycle-cyclist geometries, respectively.

A Sense 3D scanner (3D Systems, Inc., Rock Hill, SC, USA ) with the respective software (Sense, 3D Systems, Inc., Canada) allowed for obtaining the geometry with the subject in upright position [15]. The geometry was edited on the Geomagic Studio software (3D Systems, USA) and converted in CAD models [16]. Then, in the same software, two new CAD models were created as amputated cyclists. The editions were to create bicycle-cyclist system geometries for able-bodied, Tr and Tt (Figure 1).

### 2.2. Boundary Conditions

The three-dimensional boundaries with 7 m of length, 2.5 m of width and 2.5 m of height were created in Ansys Workbench software (Ansys Fluent 16.0, Ansys Inc., Pennsylvania, PA, USA) around the bicycle-cyclist system for each geometry. The Ansys meshing module allowed to generate a grid with more than 42 million of elements to represent the fluid flow in the opposite direction to the bicycle-cyclists systems at 2.5 m distance of the fluid flow inlet portion [17].

Mean velocity in tours is near 11.1 m/s (~40 km/h) [28,29]. Knowing that, velocities up to 13 m/s with increments of 1 m/s. The velocities were set at the inlet portion of the enclosure surface (-z direction) in the opposite direction of the bicycle-cyclists models’ orientation. The turbulence intensity in numerical simulations were assumed as 1 × 10^−6^%. It was established that the bicycle–cyclist system had a zero roughness non-slip wall, and scalable wall functions were assigned.

### 2.3. Numerical Simulations

The Fluent CFD code (Ansys Fluent 16.0, Ansys Inc., Pennsylvania, PA, USA) solve the Reynolds-averaged Navier–Stokes (RANS) equations by the finite volume approach to. The Realizable k-e turbulence model was selected.

For pressure–velocity coupling, the SIMPLE algorithm was used [16]. The discretization schemes were defined as second for the pressure interpolation and the convection and viscous terms. The gradients were computed by the least-squares cell-based method. Pressure and momentum were defined as second-order and second-order upwind. The turbulent kinetic energy and dissipation rate were defined as first order upwind. The convergence occurred automatically by the Ansys Fluent 16.0 before 1404 interactions.

### 2.4. Outcomes

#### 2.4.1. Drag Force

The coefficients of drag and effective surface were obtained from the numerical simulations (Ansys Fluent 16.0, Ansys Inc., Pennsylvania, PA, USA). The drag force was computed by Equation (1)

(1) $$FD=0.5\rho AC_{d}v^{2}$$

*FD* is the drag force, *C_d_* represents the drag coefficient, *v* the velocity, *A* the surface area and *ρ* is the air density (1.292 kg/m^3^).

#### 2.4.2. Energy Cost

Knowing drag and rolling resistance, Equation (2) enables the assess of the energy cost (i.e., energy expenditure per unit of distance) [14].

(2) $$Ec=\frac{CR.m.g+\frac{\rho }{2}.A.C_{D}.v^{2}}{n}$$

In Equation (2), *Ec* is the energy cost, *CR* is the rolling coefficient, *m* the body mass of the bicycle-cyclist system, *g* the gravitational acceleration, *v* the mean velocity over the race, *ρ* the air density, *A* is the surface area and *CD* the drag coefficient and *η* the gross efficiency. The assumed gross efficiency of cyclists is 20% [29] and *CR* 0.00368 [14].

The body mass was estimated based on body segment parameter of Zatzyorsky adapted by Leva [30]. Thus, the subject with Tr amputation might weight 64.28 kg and with Tt 63.15 kg.

### 2.5. Statistical Analysis

The normality and homoscedasticity assumptions were analyzed by Kolmogorov–Smirnov and Levene tests, respectively. One-way ANOVA was used to test a hypothetical variation in the drag and energy cost among the three cyclists, at different speeds. Afterwards, whenever suitable, Bonferroni test compared differences between pairwise cyclists. Effect sizes were computed based on eta squared (*η*^2^), and interpreted as [31]: without effect if 0 < *η*^2^ ≤ 0.04, minimum if 0.04 < *η*^2^ ≤ 0.25, moderate if 0.25 < *η*^2^ ≤ 0.64 and, strong if *η*^2^ > 0.64.

Simple linear regression models between able-bodied, Tr and Tt for absolute values and after square root transformation (√) were used [32]. A trendline equation, determined the correlation coefficient (*R*^2^). Effect sizes were deemed as very weak if *R*^2^ < 0.04, weak if 0.04 ≤ *R*^2^ < 0.16, moderate if 0.16 ≤ *R*^2^ < 0.49, high if 0.49 ≤ *R*^2^ < 0.81 and very high if 0.81 ≤ *R*^2^ < 1.0 [27,28,33]. Moreover, reference lines were included in plots.

## 3. Results

The one-way ANOVA showed no statistical variations between models in the drag force (able-bodied vs. Tr vs. Tt) (*F* = 0.041; *p* = 0.960; *η*^2^ = 0.002) and in the energy cost (*F* = 0.42; *p* = 0.908; *η*^2^ = 0.002).

**Figure 2:**
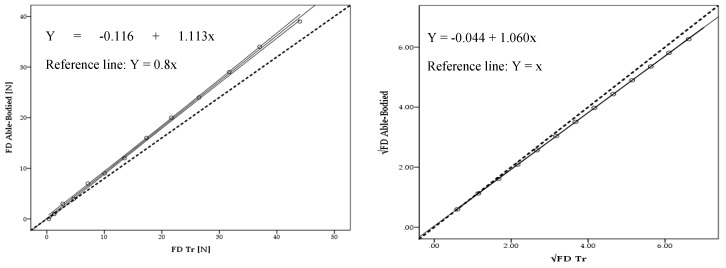
Scattergram, CI lines, tendency line (black) and reference line (dashed black) between able-bodied and Tr model for absolute drag (FD) values (**left**) and after square root (√) transformation (**right**).

The able-bodied mean drag (FD) over the 13 selected velocities was 15.23 ± 13.02 N, whereas, the Tr was 16.76 ± 13.62 N (Δ = 9%). Drag presented no statistical variations between models (*F* = −1.53; *p* = 1.00; *η*^2^ = 0.003). The linear regression models between able-bodied and Tr showed a very high adjustment for drag in absolute values (*R*^2^ = 1.000; *Ra*^2^ = 1.000; *SEE* = 0.20) (Figure 2 left panel) and after square root transformation (*R*^2^ = 1.000; *Ra*^2^ = 1.000; *SEE* = 0.008) (Figure 2 right panel).

**Figure 3:**
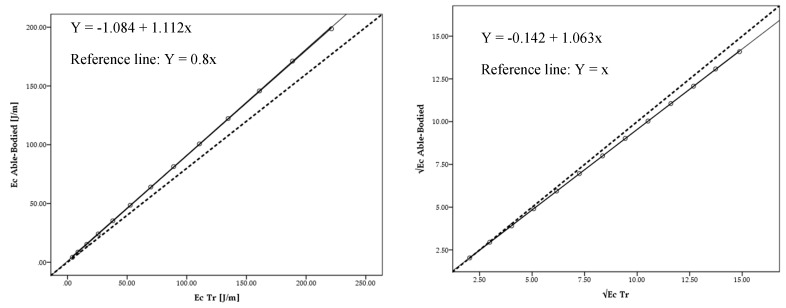
Scattergram, ICC lines, tendency line (black) and reference line (dashed black) between able-bodied and Tr for absolute energy cost (Ec) values (**left**) and after square root (√) transformation (**right**).

The mean energy cost (Ec) for the able-bodied was 78.44 ± 64.74 J/m, whereas for the Tr was 86.17 ± 72.02 J/m (Δ = 9%). Energy cost showed no statistical variations (*F* = 0.42; *p* = 0.959; *η*^2^ = 0.003). The linear regression models between able-bodied and Tr presented a very high adjustment for energy cost for absolute values (*R*^2^ = 1.000; *Ra*^2^ = 1.000; *SEE* = 0.57) and after square root transformation (*R*^2^ = 1.00; *Ra*^2^ = 1.00; *SEE* = 0.017). The absolute values differences magnitude between the able-bodied and Tr are presented in Figure 3 (top right and bottom right panels).

**Figure 4:**
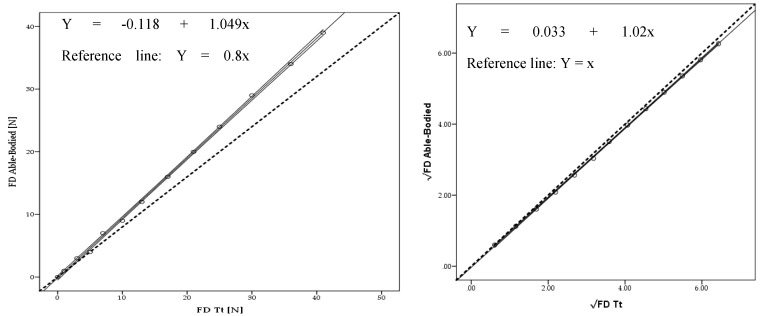
Scattergram, ICC lines, tendency line (black) and reference line (dashed black) between able-bodied and Tt model for absolute drag (FD) values (**left**) and after square root (√) transformation (**right**).

The Tt model mean drag across the 13 selected velocities was 16.08 ± 13.62 N. The able-bodied subject was 15.23 ± 13.02 N. Drag presented no statistical differences (*F* = −0.85; *p* = 1.00; *η*^2^ = 0.001) between the two models (Δ = 5%). The linear regression models between the able-bodied and Tt presented a very high adjustment for drag absolute values (*R*^2^ = 1.00; *Ra*^2^ = 1.000; *SEE* = 0.16) (Figure 4 left panel) and after square root transformation (*R*^2^ = 1.00; *Ra*^2^ = 1.00; *SEE* = 0.011) (Figure 4 right panel).

**Figure 5:**
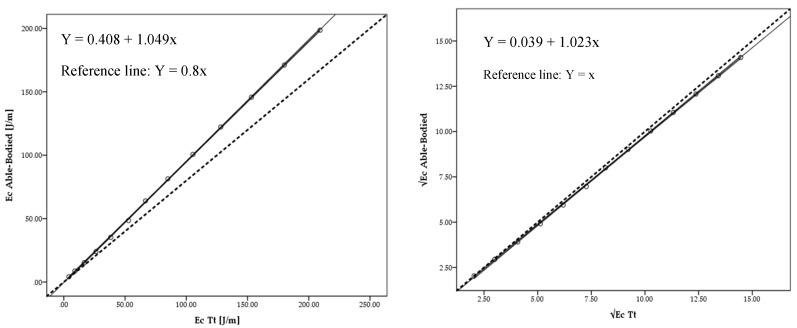
Scattergram, ICC lines and tendency line (black) and reference line (dashed black) between able-bodied and Tt model for absolute (Ec) values (**left**) and after square root (√) transformation (**right**).

The mean energy cost for the able-bodied was 78.44 ± 64.74 J/m; whereas for the Tt was 82.67 ± 67.90 J/m (Δ = 5%). Energy cost had no statistical difference (*F* = −4.23; *p* = 1.00; *η*^2^ = 0.001). The linear regression models between able-bodied and Tt presented a very high adjustment for energy cost for absolute values (*R*^2^ = 1.00; *Ra*^2^ = 1.00; *SEE* = 0.778) (Figure 5 left panel) and after square root (√) transformation (*R*^2^ = 1.00; *Ra*^2^ = 1.00; *SEE* = 0.062) (Figure 5 right panel).

## 4. Discussion

This study aimed assess and compare three cyclist’s energy cost assessed by CFD and analytical procedures, with Tr and Tt amputation with an able-bodied at different speeds. Drag and energy cost were assessed base on numerical simulations and analytical procedures, respectively. This study allowed concluding that an able-bodied cyclist had lower drag and energy cost, followed by the Tt and Tr cyclists.

The mean drag for able-bodied was 15.23 ± 13.02 N, 16.76 ± 13.62 N for the Tr and 16.08 ± 13.62 N for Tt. No statistical variations were founded across the three cyclists (*F* = 0.041; *p* = 0.960; *η*^2^ = 0.001). The drag difference between the able-bodied and Tr was 9% and 5% between the Tt and able-bodied cyclists. At least one piece of research was found, studying an uni-lateral trans-tibial amputation wearing two different prosthesis [11]. The authors [11] reported effective surface area (product between surface area and drag coefficient: ACd) values of 0.246 and 0.253 m^2^ for speeds near 12 m/s in a uni-lateral trans-tibial amputation wearing two different prosthesis systems. Thus, drag values at cyclists mean speed might be between 21.66 and 22.27 N. In our study, at the selected speed, drag values for a uni-lateral trans-tibial amputation were between 28.68 and 31.71 N, where the able-bodied presented lower drag, followed by the Tt and the Tr. The results of our study were slightly larger, due to: (i) in the abovementioned study, the cyclist was in a time trial position, whereas, in our study the cyclist was in the upright position; (ii) the authors assessed drag in a wind tunnel, conversely, in our study, we have used numerical simulations. No study was founded on drag in forearm-amputated cyclists. However, with an able-bodied cyclist and for the same position, some studies present ACd values between 0.37 m^2^ and 0.42 m^2^ [8,34,35,36]. For 11 m/s (near cyclists mean speed) drag might range between 28.92 and 32.83 N [8,34,35,36]. In our study and in the same conditions, drag ranged between 28.68 and 31.71 N. The Tt and Tr presented higher values in comparison to the able-bodied. That might be explained by the generated vorticity around the bicycle–cyclist system, increasing drag. As such, follow-up studies are suggested, focusing on the fluid flow around the cyclist with and without prosthesis or different types of prothesis.

Linear regression presented a very high adjustment for drag for absolute values (*R*^2^ = 1.000; *Ra*^2^ = 1.000; *SEE* = 0.20) and after square root transformation (*R*^2^ = 1.000; *Ra*^2^ = 1.000; *SEE* = 0.008) between able-bodied and Tr. Moreover, between the able-bodied and Tt a very high adjustment was also founded for drag absolute values (*R*^2^ = 1.00; *Ra*^2^ = 1.000; *SEE* = 0.16) and after square root transformation (*R*^2^ = 1.00; *Ra*^2^ = 1.00; *SEE* = 0.011). To date, no study has been found comparing drag and energy cost between able-bodied and Tr and Tt amputees. However, it is possible to find some studies assessing bias between methods with this statistical analysis [26,27]. Based on this procedure, it is possible to reinforce the possibility to assess amputated cyclists drag based on their counterparts.

The mean Ec for the able-bodied was 78.44 ± 64.74 J/m, for the Tr cyclist was 86.17 ± 72.02 J/m and for the Tt 82.67 ± 67.90 J/m. The Ec difference between the able-bodied and Tr was 9% and 5% between the Tt and able-bodied. To our understanding, this is the first attempt to estimate amputated cyclist energy cost based on numerical simulations and analytical procedures in amputees. No study was found reporting amputated cyclist energy cost. However, in able-bodied counterparts and with maximal effort, in a laboratory ergometer bicycle with a constant power output of 150 W, the energy cost ranged between 1.11 and 2.39 J/m/kg [37]. Thus, considering 65 kg of mass, the energy cost might be 72.15 and 155.35 J/m [37]. These values are in accordance with our research. Moreover, in long distances and considering the cyclists’ mean speed (≈11 m/s: 40 km/h) the energy cost is about 100 J/m in elite cyclists participating in the Tour the France [13]. Once more, these results show good adherence to our data. The difference might be due to: (i) between-subjects differences between the participant of this study and Tour the France cyclists; (ii) the methods selected to assess Ec were different.

The linear regressions presented a very high adjustment for absolute values in Ec between able-bodied and Tr (*R*^2^ = 1.000; *Ra*^2^ = 1.000; *SEE* = 0.57) and Tt (*R*^2^ = 1.00; *Ra*^2^ = 1.00; *SEE* = 0.778) and after square root transformation (*R*^2^ = 1.00; *Ra*^2^ = 1.00; *SEE* = 0.017) and (*R*^2^ = 1.00; *Ra*^2^ = 1.00; *SEE* = 0.062). To our understanding, there is no study reporting these data in the literature. However, the adjustments between drag may predict Ec adjustment (Equation (2)) [6].

Altogether, this study concluded that the able-bodied cyclist presented less drag and energy cost, followed by the Tt and Tr amputee. This paper also presented a correction factor to estimate Tr and Tt drag and energy cost.

This study presents the following limitations: (i) only one cyclists was assessed and the simulations were made for amputees without prothesis; (ii) only one single position was assessed.

## 5. Conclusions

This study allowed concluding that drag and energy cost were lower on the able-bodied, followed by the Tt and Tr amputees. Variations with no effects were founded in drag and energy cost across able-bodied, Tr and Tt cyclists. Moreover, a very high adjustment for drag and energy cost was verified, suggesting the possibility of using these data to analyze different body disabilities.
